# A comparative analysis of cancer stage classification systems for registries

**DOI:** 10.3332/ecancer.2025.1920

**Published:** 2025-06-03

**Authors:** Abhinav Ramraj, Hariharasudhan Saravanan

**Affiliations:** Department of Community Medicine, Stanley Medical College, Chennai 600001, India; a https://orcid.org/0009-0002-9621-3248; b https://orcid.org/0009-0008-6029-9610

**Keywords:** neoplasm staging, registries, epidemiological monitoring, data collection, records

## Abstract

Cancer stage at diagnosis is a critical determinant of survival outcomes and a key metric for population-based cancer surveillance. Despite the existence of several cancer staging classifications implemented in registries worldwide, their relative utility remains poorly understood. This review provides a comprehensive and comparative evaluation of the principles, data requirements and practical utility of the traditional tumor-node-metastasis (TNM), Surveillance, Epidemiology and End Result Summary, Condensed TNM, Essential TNM, registry-derived and extent-of-disease staging systems. It also introduces a conceptual framework for evaluating these systems, in order to aid registries in selecting context-appropriate staging methods. Our appraisal, focusing primarily on aspects pertaining to data collection and consolidation, recognises that while the traditional TNM system offers the highest clinical and prognostic value, its complexity leads to poor completeness in population-based registries, particularly in low- and middle-income countries. Simplified alternatives can achieve higher completion rates but offer limited clinical utility. A balanced approach jointly incorporating clinical value and practical feasibility is essential, highlighting the need for hybrid solutions to support cancer registration. Electronic aids such as staging applications and natural language processing or AI-driven tools can streamline staging by automating data extraction, minimising errors and inferring missing components. Future efforts must prioritise accessible, multilingual platforms to standardise surveillance and improve accuracy in resource-limited settings.

## Background

Cancer incidence continues to rise, predominantly affecting low- and middle-income countries which have higher incidence and mortality rates. Devising, implementing and monitoring cancer control strategies necessitate reliable real-world data on cancer incidence and survival at the population level. Cancer registries, especially population-based (PBCRs), are the only source of such population-level burden and outcome indicators and continue to provide timely, real-world data across diverse settings [1].

One of the most defining characteristics of cancer, proven to influence survival consistently across types, is its stage at presentation. Cancer stage classifies the extent or ‘spread’ of the disease and has both clinical and epidemiological significance; the stage at diagnosis determines the most suitable treatment approach and is also an established and strong correlate of survival. Recording the stage of cancer cases, ideally at the population level, is not only necessary to estimate stage-specific survival and evaluate the efficacy of healthcare systems, but is also crucial to monitor longitudinal trends in stage distributions [2–6]. This helps draw meaningful comparisons between regions or populations and assess the impact of cancer control programs. Cancers are staged using criteria defined by classification systems which categorise patients with similar anatomic extents of disease into discrete and ordinal categories or ‘stage-groups’. Cases within a stage category are expected to have similar prognosis and management, hence necessitating such classifications to undergo periodic and evidence-based revision based on real-world clinical data to better convey prognostic value through their categories.

Although lacking granular treatment-related details, PBCRs cover well-defined geographic and demographic regions and aggregate data from a wide range of sources (field, hospitals, diagnostic centers and so on) in their designated jurisdictions [7–9]. By offering a comprehensive overview of incidence, prevalence and survival trends at the population level, they continue to remain essential for understanding cancer burden and outcomes in regional public health planning. On the other hand, other registry types such as hospital-based (HBCR) and tumour specific or special purpose registries offer critical insights by leveraging detailed clinical records and treatment data. These systems are often integrated with electronic health records, thus enabling more accurate stage assignments and robust outcome evaluations [10]. HBCRs operate within healthcare facilities, compiling detailed treatment information on patients diagnosed or treated at their individual facilities, and support internal patient care and quality improvement [11–13]. Special-purpose or tumour-specific registries, meanwhile, target specific aspects related to cancer care and often cater to specific tumour sites, rare cancers or niche population groups. They combine clinical data with more extensive molecular, genetic and treatment variables such as biomarkers and family histories, enabling in-depth analyses into aetiologies and therapeutic efficacies in order to enable and support focused research and personalised approaches [14–17]. Collectively these registry types complement each other by addressing different dimensions of cancer surveillance – while HBCRs offer detailed and granular, but institution-specific, insights focusing on care-related outcomes, PBCRs provide a broad population-level perspective of burden and special purpose/tumour-specific registries deliver focused data to drive specialised research and improvements in targeted cancer care.

However, collecting stage-related information at scale is a challenging endeavour for both high-income countries and low- and middle-income countries (LMICs) [18]. While PBCRs across the globe have generally lacked stage-related information, with several not even attempting to capture stage data [19, 20], the data that does exist is often incomplete and of insufficient quality for analysis [6, 18, 21]. The extraction of TNM data poses significant challenges in LMICs, where infrastructural constraints and fragmented healthcare systems amplify existing complexities. Unlike high-income settings, LMICs often lack integrated health information systems, forcing registries to rely on disparate data sources such as clinical records, pathology reports and imaging findings, which are frequently inconsistent or incomplete [22]. These sources are often spread across multiple facilities, creating a fragmented data landscape that spans long periods of the clinical care continuum. In many instances, clinicians in resource-constrained settings may fail to explicitly document the TNM components in medical records, either due to time constraints, lack of awareness or the absence of standardised reporting protocols [23–25]. This leaves critical details to be inferred, which in turn forces registrars—who may lack clinical backgrounds—to interpret ambiguous narrative descriptions, leading to errors and misclassification. Furthermore, the lack of centralised electronic health records (EHRs) and reliance on paper-based systems without structured reporting formats, exacerbates these issues as free-text narratives can omit critical details like nodal involvement or metastasis status, hinder timely data retrieval and increase the risk of data loss. In many LMICs, limited access to advanced diagnostic tools, such as PET-CT scans or molecular testing, further restricts the ability to accurately determine metastatic or nodal status. Addressing these challenges requires targeted investments in health infrastructure, standardised data collection and reporting protocols [25, 26] and capacity-building initiatives to ensure accuracy in point-of-care settings [27, 28].

Moreover, even if data collection is effective, the lack of a standardised approach to assigning cancer stage leads to registries within and across countries reporting stage assigned on the basis of different criteria. Several staging systems exist – the TNM system, the Surveillance, Epidemiology and End Result (SEER) Summary stage, Essential TNM (ETNM), Condensed TNM (CTNM) and the degree of spread systems. Although currently in use in registries worldwide, they are poorly understood, with little published literature detailing the nature of the categorisation offered. They differ based on their intended application, clinical relevance and categorise in a manner that often cannot be compared with each other. This hinders comparability and harmonisation of data for epidemiological or outcomes analyses and benchmarking studies within health systems, regions and between countries. Moreover, it is unlikely that we achieve international consensus in the near future [29] and registries continue to record and report stages using varying systems, with high-income countries like the United States, Canada, United Kingdom and in Europe, reporting in more than one[21, 22, 29–31].

The lack of a conceptual framework comparing these systems prevents registries, especially those in LMICs, from critically appraising the various classification schemes and determining the system(s) and approaches best suited to their epidemiological needs and data collection methods. This review begins with a brief overview of the classification systems and later offers a comparison from the perspective of data collection and consolidation. The detailed tumour-specific criteria can be found in the latest versions of the corresponding manuals [18, 32–34] but are not described here.

## Cancer stage classification systems

**Figure 1.**
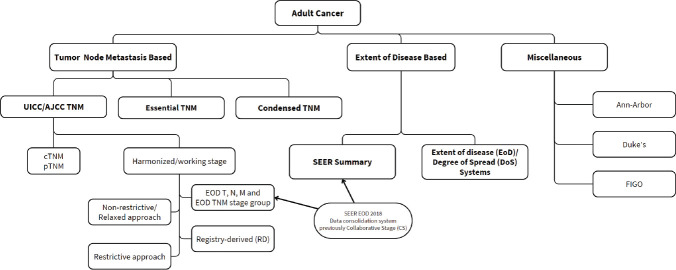
Cancer stage classification systems and approaches to data consolidation.

Staging classification systems have been designed for diverse settings at different periods in time and with different intended applications. They primarily vary in the nature and depth of information required to assign a particular case of cancer to a stage category. The oldest and most widely implemented system, in clinical practice and in registries across the globe, the Union for International Cancer Control (UICC)/ American Joint Committee on Cancer (AJCC) TNM system (hereafter referred to as just the TNM system) has been associated with high rates of incompleteness [20, 29, 31]. Recognising the challenges in collecting TNM stage information, several simplified classifications and approaches to reconciling missing data have since been developed, which are summarised in Figure 1.

## UICC/AJCC TNM

The tumour, node and metastasis (TNM) staging system developed in the mid-20th century and currently maintained by the UICC in collaboration with the AJCC has remained the global standard for assessing cancer extent for over 75 years. Although these are separate organisations that publish their own manuals, their goal is to maintain identical classifications and any variations that may exist are reconciled in subsequent editions, the latest of which was published in 2017 [18, 32]. However, despite shortcomings, and in view of its intuitive underlying principle and the consistent and strong association between the TNM stage and patient outcomes, it remains the most widely followed by clinicians and registries worldwide.

## Condensed TNM

CTNM was developed by the European Network of Cancer Registries (ENCRs) in 2002 as a simplified alternative to the traditional TNM system [34, 35]. It is based on the same T, N and M components but uses general criteria applicable to all tumour types, utilising both clinical and pathological TNM and/or descriptive information. Since its inception in 2002, however, the guidelines have not been revised and it has not been widely adopted by European registries which seem to prefer traditional TNM instead [21].

## Essential TNM

The ETNM staging system, a collaborative effort by the UICC, the International Agency for Research on Cancer (IARC), and the International Association of Cancer Registries, was designed for use when complete TNM data is unavailable, especially for registries in LMICs. It aims to enable stage assignment with lesser data but in a manner comparable with the TNM stage categories [20, 36]. However, it has been deemed to require further field-testing and dissemination programs [20] and has not yet been officially implemented in registries.

## Registry-derived stage

The registry-derived (RD) stage emerged as a response to the lack of standardised stage data in Australian registries through the national stage, treatment and recurrence project, initiated in 2011 by Cancer Australia in collaboration with the Australian Institute of Health and Welfare and state and territory cancer registries [37, 38]. This approach represents an important step forward in standardising cancer stage data consolidation and has proven to be a valuable tool in addressing notable gaps in Australia’s national cancer data; it has significantly improved completeness of staging data across various cancer types enabling the country’s first national-level reporting of cancer stage at diagnosis for the top five incident cancers [38].

## SEER Summary 2018

The SEER Summary Stage (SSS) was initially developed by the National Cancer Institute’s End Results Group in the 1950s for the SEER Program, an authoritative source of cancer stage data in the United States [30]. It continues to remain an integral part of reporting in the SEER Program and is currently (going forward from January 2018) mandated in central registries in the United States [39, 40].

## Miscellaneous

Extent of disease (EoD) systems are locally developed by and tailored to meet the needs of specific PBCRs. For example, the New South Wales Cancer Registry (NSWCR) uses the ‘Degree of Spread (DoS)’ system for all non-hematopoietic cancers on the basis of criteria defined by the IARC [41]. Tumour-specific classification systems, such as Ann–Arbor for lymphomas and Duke’s for colorectal cancer, exist but these are largely not relevant in the context of data consolidation in registries.

The Toronto Guidelines address the unique biological and clinical features of childhood malignancies and provide a pragmatic, consensus-based framework for staging [42, 43]. Emphasising clinical, imaging and histological criteria for risk stratification and clinical decision-making, these guidelines harmonise data collection across institutions to improve comparability in survival analyses and facilitate collaborative research. The Guidelines advocate for type-specific staging criteria for 16 of the most prevalent paediatric malignancies and has been deemed optimal for PBCRs by the IACR and ENCR [44, 45]. Moreover, they delineate a two-tiered framework whereby registries constrained by limited resources or data access are provided with less granular criteria (Tier 1), whereas those with greater resources are afforded more detailed criteria (Tier 2) [42, 44, 46]. To ensure comparability across registries, the stage categories defined under Tier 2 may be consolidated to correspond with those of Tier 1.

## Comparative analysis of cancer stage classifications

**Figure 2.**
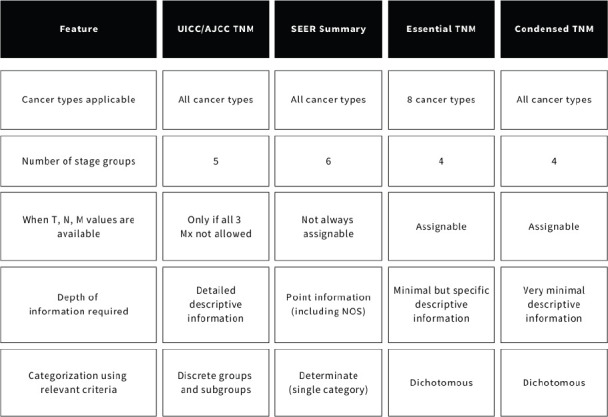
Comparing the cancer stage classifications from the perspective of data collection and consolidation.

A comparative analysis of their underlying principles, classification schemes and depth of information required to stage cases, with a focus on data collection and consolidation within registries, is presented under the following headings and summarised in Figure 2.

## Categorisation scheme

The TNM system has specific criteria for each cancer type, primarily based on the anatomic extent, with the core components being tumour size and/or extent (T), lymph node involvement (N) and metastasis (M). After assigning values for T, N and M using the defined criteria, cases are grouped into one of several disparate but tiered categories; allowing for ‘telescoping’ [18] between either broader stage groups 0, I, II, III and IV or narrower distinctive subcategories within these groups (Ia1, IIb and so on), based on the depth of available information. It also recognises the source of, and point during care the relevant information is obtained, by distinguishing between clinical, pathological, post-therapy and recurrence staging.

CTNM and ETNM, however, while retaining the core T, N and M components, employ a largely dichotomous categorisation; localised and advanced for tumour size/extent (L versus A in CTNM and L/L1/L2 versus A/A1/A2 in ETNM), nodes (N0 versus N+ in CTNM and R- versus R1/R2 in ETNM) and metastasis (M0 versus M+ in CTNM and M- versus M+ in ETNM). The resultant stage categories are fewer but broader – tumour localised (TLN0M0), tumour with local spread (TAN0M0), tumour with regional spread (any T N+ M0) and advanced cancer (any T any N M+) in CTNM. In ETNM, the categories – localised limited (L R- M-), localised advanced (A R- M-), regional limited or extensive (L/A R+ M-), to distant metastasis (L/A any R M+), are also designed to correlate with the traditional TNM stage groups (I-IV) [33], but with the relative position of localised advanced with regional interchanging based on cancer type.

RD staging, on the other hand, is not a classification system, but rather a country-specific approach to deriving stage for population-based analyses from routine sources where TNM data is incomplete [22]. It uses simplified business rules developed by the Victoria Cancer Registry [38, 41, 47] which articulate the decision-making process for defining the RD-stage categories in alignment with the TNM stage groups defined in the AJCC 7th Edition. The stage is reported I-IV, but the detailed business rules used for assignment are currently unavailable on their website or elsewhere in published literature.

SSS represents a fundamental approach to categorisation quite distinct from the TNM-based systems. It employs a hierarchical one-digit coding system with six categories, but can be further condensed into three broad levels: localised, regional and distant [39]. Unlike TNM, where tumour size and number and pattern of nodal involvement are crucial criteria, SSS disregards either and only concerns with the organ(s) involved and follows the usual dichotomy for nodal involvement and metastasis; defaulting to code 3 when nodes are involved and code 7 when metastasis is present, regardless of primary tumour extent.

EoD/DoS systems classify tumours broadly as localised, regional or metastatic, (but sometimes as hybrid categories like L/R or R/M when uncertain) as well, but use unclear criteria that varies among registries [21, 22] and which are not published.

## Data complexity and ease of data consolidation

The TNM system is the most comprehensive, offering granular and detailed criteria while also explicitly providing the range of ICD-O3 codes the classifications are applicable to, preventing ambiguity. However, it necessitates considerable detail on specific and ineludible aspects on disease extent to assign a stage. Assigning a stage group often requires all three (T, N, M) values and the source of information (clinical *vs.* pathological) and missing any crucial information may render assignment impossible. To deal with partial information and yield data sufficient for surveillance purposes, it does allow for combining clinical and pathological information to determine the ‘harmonized’ [48] or ‘working stage’ [18]. While a ‘restrictive’ approach to data consolidation requires all three values, a ‘non-restrictive’ or ‘relaxed’ approach assumes missing N or M values as zero to derive the final stage variable [6, 22, 29]. Attempts to include information from other systems (like Condensed TNM and degree of spread) as a substitute for missing TNM information to derive a ‘reconstructed stage’ have also been made [21].

ETNM prevents the need for exhaustive searches by advocating a logical decision process in the order from M >N >T (and stage groups IV> I) enabling cancer registrars to record the furthest extent of disease first, allowing them to stop searching for additional information [20, 36], but this can be applied for CTNM as well. While N and M are assigned purely on the basis of presence in both CTNM and ETNM (although N is differentiated as R1, regional limited and R2, regional extensive in ETNM, they do not contribute to a difference in the final stage category, which codes both as regional), T is determined by convention (usually T1-T2 for L and T3-T4 for A) in CTNM, whereas specific criteria that exist in traditional TNM are used for T in ETNM. However, as ETNM includes certain criteria found in the TNM classification deemed important, specific information clarifying them are required without which assignment is not possible, ensuring greater intrinsic clinical value, although making ETNM harder to achieve completion in as compared to CTNM.

RD staging uses individual TNM values or the TNM stage group if available, but a key feature is its ability to generate stage by leveraging data supplementation from secondary sources, (hospital morbidity data collections, pathology reports or clinical documentation) when assumptions are required due to data limitations [41]. Hence, data consolidation requires manual corroboration and can be time- and resource-intensive, and as it relies on assumptions and generalisations, may potentially lead to errors in staging.

SSS utilises clinical, pathological and radiological information primarily from narrative descriptions in medical records, pathology and imaging reports. SSS also provides explicit provision for less detailed information by including the ‘not-otherwise specified, NOS’ suffix within its categories. Furthermore, it employs a relaxed approach to unknown nodal or metastasis status by treating missing information as negative, which may lead to potential under-staging compared to TNM. Even isolated or unclarified and pointed information, as is often present in manual medical records or diagnostic reports, is sufficient for registrars to assign a stage, thus offering a determinate categorisation without any scope of ambiguity or need for in-depth knowledge of anatomy. The single-page coding scheme for each cancer type further supports its ease of use, especially for non-health professionals. SSS is not only less complex and easier to learn and use than the TNM system but is also updated much less frequently, not changing with revisions to the TNM system. However, it is not directly comparable with the TNM systems even when all T, N and M values (let alone the stage groups) are known. For example, as seen in the case of lip and oral cavity cancers, T information cannot be used to differentiate between codes 1 and 2 without additional information due to incomparable criteria used [39]. While it can use TNM values when it is the only data available, SSS prioritises narrative data, especially when such discrepancies arise, thus necessitating intensive manual corroboration.

EoD/DoS systems employ the same localised-regional-distant classification scheme as SSS [41], sourcing information from multiple sources, but rely on a subjective assignment to their categories, unlike SSS which explicitly lays down all possible terms clarifying disease extent to definitive stage categories. Since the criteria used by these registries are often unclear, comparisons or mapping with the TNM and other staging systems are not possible.

## Utility and limitations

The UICC/AJCC TNM criteria undergo periodic evidence-based revisions based on newly acquired clinical data and advances in the understanding of cancer biology and prognosis by a dedicated international multi-disciplinary team, ensuring its consistency across global healthcare settings [18]. Hence the TNM system remains the most clinically useful classification offering the greatest prognostic value. Nevertheless, due to the complexity and specificity of information required, completeness of T, N, M or stage group data remain low in registries across the world, especially in LMICs [20, 29, 49]. Moreover, it has increasingly begun including non-anatomic prognostic factors (such as PSA for prostate, receptor status for breast and p16 positivity for oropharyngeal cancers) to better convey clinical and prognostic value across categories [18, 32]. These additional diagnostic data points are harder to perform and collect at scale, especially in LMICs with resource and infrastructure constraints, and can be expected to make complete staging information even harder to achieve for their registries [48]. There is also inconsistent and even incorrect use of terminology related to staging with this system, different editions or approaches being used without being recorded and pooling of stage at diagnosis with stage assessed at other points of care [29, 48]. While some registries only had the stage group and not the individual TNM variables, others combined and/or did not record the source (clinical *vs.* pathological) of information used to assign the final stage variable [29]. Registries often did not possess the edition of TNM classification that was used to assign variables as this was often not recorded in the medical record or pathology reports [29]. Hence, this system may require adaptation for use in registries and simpler systems are rightly considered.

In CTNM, due to the crude binary nature of categorisation of all three components, the resultant stage categories are extremely broad, often grouping cases with grossly differing prognoses together, thus holding minimal prognostic or clinical value. Since ETNM categories are comparable with the TNM stage groups, they offer more clinical value than CTNM. However, ETNM is currently available for only eight cancer types [33].

Although data consolidation using RD-staging is resource-intensive, with regards to prognostic value, it has demonstrated a higher agreement with the AJCC TNM stage groups compared to Degree of Spread [41, 47]. However, as it is derived solely from limited datasets excluding radiology reports and clinical correlation, may lack the nuanced details critical for individual patient management and clinical decision-making [41]. Regardless of its limitations, devising country- or registry-specific business rules suited to existing datasets and data collection pipelines presents the most likely and feasible solution for missing and/or hard-to-attain TNM information.

Although the SSS classification scheme offers more granularity and specificity than other simplified systems, since it is not directly comparable to the TNM categories, it is less suitable for clinical practice or research. The relative simplicity and broad applicability has allowed high rates of completeness of up to 90% for breast and cervical cancer in the US [20, 30], whereas stability over time and TNM editions has allowed long-term trend analyses [50]. However, since SSS has been designed exclusively for use in registries [31], it remains relatively unknown outside the registry community and has been used only in some countries such as the United States, Canada, Australia and New Zealand [22, 31, 50]. Nevertheless, it is a comprehensive staging classification system that offers a reliable and validated practical approach to stage data collection and consolidation that is broad enough to monitor longitudinal trends and evaluate the success of cancer control programs and other epidemiological efforts. The authors recognise it as the only reliable alternative to the TNM system for use in cancer registries in LMICs, which have consistently faced low rates of TNM information, in the near future.

EoD/DoS systems have been adopted by PBCRs in several countries in Europe, Australia and New Zealand, either solely or in combination with other staging systems [22, 31]. The relative simplicity of these systems has facilitated high levels of completeness in the NSWCR and in some countries like Norway and has been extensively used for reporting, survival analyses and international comparisons [29, 41]. It is important to note that while the broad categories of these systems allow for easier collection and staging, yielding data for epidemiological analyses, the lack of detail limits their application for prognostication.

## Conclusion

This review offers a comprehensive and comparative appraisal of cancer stage classifications, providing registrars and researchers with a framework for independently evaluating these systems across key data consolidation characteristics. As a consequence of the complexities of cancer classification, it is evident that cancer registries are presented with two primary strategic options. They could either implement targeted strategies to improve existing TNM information abstraction or explore alternative classification systems that might better suit contextual constraints. With the continued addition of sophisticated data points reflecting non-anatomic prognostic factors, sufficient TNM information for stage categorisation using the newer versions of the criteria are expected to be even harder to attain, especially at scale and in developing nations. Considering the current global landscape which demonstrates significant variability in availability and quality, and with the lowest data contribution from LMICs, alternative strategies for deriving data for population-based analyses merit serious consideration.

By establishing a foundational subjective ontology for cancer staging, the review not only clarifies existing knowledge gaps, but also intends to encourage open discourse on methodological alternatives to established TNM-based systems for the purposes of epidemiological analysis. This review particularly directs the scope of further research by introducing new avenues for methodological refinement, wherein mapping disease extents of multiple cancer types across all available classification systems could generate more nuanced analytical insights and potentially develop sophisticated stage conversion algorithms capable of seamlessly interchanging or translating between different stage categories. The authors believe that cancer-type-specific strategies that effectively address the aforementioned challenges associated with data incompatibility can yield solutions to facilitating data harmonisation for benchmarking studies.

Nevertheless, the integration of electronic aids like staging applications and automated data retrieval tools offer transformative potential to address such challenges by consolidating and interpreting clinical data. Digital staging applications like CanStaging+ [51], AJCC Staging Online [52] and TNM Cancer Staging (Android) [53] among others, can streamline workflows by automating repetitive tasks. Such tools can not only minimise human error, but also ensure adherence to the latest classification guidelines. By enabling clinicians or even clerical assistants to quickly compute stage based on well-defined preset parameters, these tools can greatly reduce the need for laborious corroboration of retrieved clinical information to assign the T, N and M components and final stage group at the level of the registrar/data entry operator. Furthermore, these tools have the potential to be integrated with natural language processing frameworks to enable structured extraction of stage-related data from unstructured electronic medical record narratives [54–56]. Predictive tools can further enhance completeness by leveraging machine learning to infer missing staging components from existing partial clinical inputs, particularly beneficial in low-resource settings with fragmented data. Future global efforts should prioritise the development and refinement of open-access, multilingual platforms tailored to diverse healthcare infrastructures and standardise their application to maximise impact on patient outcomes and cancer control strategies. While such tools require rigorous validation [57] and adaptation to local contexts, their adoption could significantly improve staging accuracy and efficiency, especially in LMICs who are more likely to face operational constraints.

However, irrespective of the chosen system(s) and/or approaches, it is imperative to ensure that an alternate method of stage categorisation does possess demonstrable clinical value, ideally encompassing both prognostic and management dimensions. The review unequivocally reiterates that staging classifications designed solely for data collection and reporting purposes are devoid of actionable value both at the individual and population levels. Ultimately, registrars must ensure that data consolidation and harmonisation remain not merely a bureaucratic exercise, but a meaningful tool for clinical and outcomes research capable of supporting population-level survival analyses and guiding tangible cancer surveillance and control programs.

To address these challenges a nuanced and balanced approach is called for, which involves implementing periodic evidence-based revisions to incrementally enhance prognostic value while also maintaining sufficient simplicity to facilitate large-scale data collection. Moreover, any such system must provide sufficient stability to enable meaningful longitudinal stage distribution monitoring. Only a pragmatic approach can reconcile the diverse needs of multiple stakeholders – clinicians, cancer registrars, public health professionals and policy makers – ultimately contributing to more effective resource allocation and ensuring compliance with evolving cancer care standards.

## Conflicts of interest

The authors report no conflicts of interest.

## Funding

This research did not receive any financial support or funding.

## Author contributions

AR conceived and designed the study, reviewed the literature and wrote the final manuscript. HS reviewed the literature and wrote the final manuscript. Both authors approved the final manuscript.
